# Editorial: Adult neurogenesis as a regenerative strategy for brain repair

**DOI:** 10.3389/fnmol.2022.1041009

**Published:** 2022-10-26

**Authors:** Daniel A. Berg, Kyung-Ok Cho, Mi-Hyeon Jang

**Affiliations:** ^1^Institute of Medical Sciences, University of Aberdeen, Aberdeen, United Kingdom; ^2^Department of Pharmacology, College of Medicine, The Catholic University of Korea, Seoul, South Korea; ^3^Department of Neurosurgery, Robert Wood Johnson Medical School, Rutgers, The State University of New Jersey, New Brunswick, NJ, United States

**Keywords:** adult neurogenesis, brain disease, neural stem cells, hippocampus, regenerative therapy

Adult neurogenesis represents a striking example of neural plasticity throughout life where adult-born neurons are continuously generated from neural stem cells (NSCs). The hippocampus is a neurogenic niche where postnatal neurogenesis occurs, where the role of neurogenesis in this brain region is to maintain healthy hippocampal function, such as learning and memory across the lifespan (Clelland et al., 2009; Sahay et al., 2011; Christian et al., 2014). Impaired adult hippocampal neurogenesis is observed in a variety of neurological, neuropsychiatric, and neurodegenerative disorders and underlies the cognitive impairments associated with these disorders (Hussaini et al., 2014; Cho et al., 2015; Moreno-Jimenez et al., 2019). Therefore, a better fundamental understanding of the mechanisms that regulate adult hippocampal neurogenesis will identify disease pathologies driving cognitive impairment and provide an avenue to help design effective therapeutic strategies. This Research Topic aims to explore mechanistic evidence involved in adult neurogenesis in the healthy and pathological brain as well as discuss recent technical advances investigating adult neurogenesis.

During the process neuronal differentiation and maturation, NSCs/neural progenitors require coordinated changes in the pattern of gene expression. While epigenetic regulatory mechanisms have emerged as a critical regulator to fine-tune and coordinate gene expression during adult hippocampal neurogenesis (Berg et al., 2019), the exact epigenetic mechanisms responsible for neuronal differentiation are still elusive. Using a viral approach in a culture system, Nieto-Estevez et al. provided a novel epigenetic marker regulating adult hippocampal neurogenesis by demonstrating that histone deacetylase 1 (HDAC1) could control neuronal differentiation without affecting the proliferation of hippocampal NSCs. They also showed that while HDAC1 and HDAC2 was expressed in all stages of adult hippocampal neurogenesis, stem/progenitor cells exhibited a higher tendency for HDAC1 expression, whereas immature granule neurons had more HDAC2 expression. However, conditional HDAC1 knockout-mediated influence on the neuronal differentiation was not reproduced in an *in vivo* system, suggesting that more complex mechanisms are at play regarding the role of HDAC1 in adult neurogenesis.

Dysregulation of adult hippocampal neurogenesis contributes to a variety of brain diseases (Toda et al., 2019). For example, temporal lobe epilepsy (TLE) is a severe neurological disorder that disrupts the structure and function of the hippocampus. TLE is characterized by seizures in the temporal lobe and these seizures cause aberrant neurogenesis in the adult dentate gyrus (Cho et al., 2015). Anxiety is a major comorbidity of TLE, which greatly affects TLE patient quality of life (Vinti et al., 2021). Although the molecular and cellular mechanisms linking TLE with anxiety are not known, analysis of cerebrospinal fluid from patients suffering from generalized anxiety disorders and TLE have shown that both these conditions exhibit increased levels of interleukin 17A (IL-17A). Additionally, IL-17A has been linked to abnormal neurogenesis and anxiety behavior. Supporting this line of inquiry, Choi et al. made use of a pilocarpine model of TLE and IL-17A knock-out mice to examine the role of IL-17A in mediating anxiety and aberrant neurogenesis in TLE. They found that IL-17A KO mice showed reduced anxiety-related behavior when examined 42–43 days after pilocarpine injection. Interestingly, electroencephalogram (EEG) recordings showed no differences in the formation of spontaneous recurrent seizures in the two groups, showing that IL-17A does not play a major role in epileptogenesis. Using histological methods, the authors found that IL-17A KO mice showed reduced neuronal death and fewer ectopic granular cells in the hippocampus without a difference in cell proliferation or number of immature neurons in the subgranular zone of the dentate gyrus. Together, their results indicate a role of IL17-A in mediating TLE-associated anxiety behavior possibly by reducing ectopic neurogenesis and neuronal death in the hippocampus.

Advances in sequencing technologies has allowed to transcriptionally distinguish populations of cells involved in adult neurogenesis (Berg et al., 2019; Cebrian-Silla et al., 2021). In particular, transcriptomic analysis techniques, such as bulk or single-cell RNA sequencing (scRNA-seq) are steadily becoming efficient ways to ascertain changes in individual populations of neural progenitor cells during homeostasis or after injury. With these advancements in technology and the cost of scRNA-seq decreasing, it is becoming a more commonly used technique. However, a caveat of employing scRNA-seq when studying adult neurogenesis is that the number of NSCs is very low compared to neurons and other differentiated cells. Due to the low RNA input obtained from scRNA-seq, it is difficult to differentiate between NSCs and transcriptionally similar cell types such as astrocytes. Bulk RNA-seq, on the other hand, is able to sequence samples with more depth, although the isolation of NSCs from tissue requires the pooling of multiple animals, which can be problematic if the generation of the animal is labor intensive. To examine the differences in results obtained from bulk and scRNA-seq of adult hippocampal NSCs, Denninger et al. compared the differentially expressed genes (DEGs) in a model of oxidative stress using both sequencing paradigms. They found that both methods resulted in little overlap in common DEGs, and the DEGs identified by sc-RNAseq were from genes that had relatively higher transcript counts and had smaller fold changes compared to the DEGs identified by bulk seq. The authors then took advantage of their two data sets of the same population of cells to develop a workflow that can determine what input of RNA is necessary to preserve unbiased detection of DEGs. Using this approach, the authors were able to determine an RNA-input threshold that generated transcriptional profiling results of NSCs comparable with bulk sequencing. This approach is a great resource for researchers studying rare cell types *in vivo*.

This Research Topic also discussed the role of L-serine as a potential neuroprotective agent for neurological disease and brain injury (Ye et al.). L-serine is an indispensable neurotrophic factor and a precursor for neurotransmitters. While the significant role of L-serine in preventing neuroexcitotoxicity, neuroinflammation, and cerebral blood flow associated with brain injuries (e.g., ischemia, stroke, and TBI) are comprehensively discussed, this review also highlights L-serine's role in adult neurogenesis and oligodendrogenesis as potential neuroprotective mechanisms. For instance, L-serine promotes the survival, proliferation and differentiation of NSCs. In addition, L-serine also stimulates the proliferation and survival of oligodendrocytes derived from NSCs following brain injury, which plays an important role in myelin recovery. Collectively, L-serine serves as a potential regenerative agent that stimulates adult neurogenesis as well as oligodendrogenesis, and may represent a therapeutic option to facilitate brain repair.

Taken together, this Research Topic provides an update on new mechanistic evidence in regulating adult neurogenesis and perspective of novel regenerative strategies to restore lost brain function in brain injury. Although extensive studies have made significant advances in mechanisms that control each developmental stage of adult-born neurons, there are still many outstanding questions and thus further studies are needed.

## Author contributions

All authors listed have made a substantial, direct, and intellectual contribution to the work and approved it for publication.

## Funding

This work was supported by Biological Sciences Research Council (BBSRC) BB/W008068/1 to DB, National Research Foundation of Korea (NRF) (2019R1A2C1003958 and 2021R1A4A5028966) to K-OC, and NIH (R01CA242158 and R01AG058560) to M-HJ.

## Conflict of interest

The authors declare that the research was conducted in the absence of any commercial or financial relationships that could be construed as a potential conflict of interest.

## Publisher's note

All claims expressed in this article are solely those of the authors and do not necessarily represent those of their affiliated organizations, or those of the publisher, the editors and the reviewers. Any product that may be evaluated in this article, or claim that may be made by its manufacturer, is not guaranteed or endorsed by the publisher.
